# Diagnostic Value of Serum GREM1 Levels in Differentiating Benign and Malignant Subsolid Pulmonary Nodules: A Prospective Observational Study

**DOI:** 10.1002/kjm2.70315

**Published:** 2026-11-09

**Authors:** Yi‐Yun Pan, Wen Zeng, Shuo Wang, Qi Wang, Hai‐Long Chen, Neng‐Ping Luo, De‐Chang Xu, Kui‐Yuan Wang

**Affiliations:** ^1^ Department of Oncology, Ganzhou Cancer Hospital Gannan Medical University Ganzhou Jiangxi China; ^2^ Department of Surgical Oncology, Ganzhou Cancer Hospital Gannan Medical University Ganzhou Jiangxi China

**Keywords:** benign, diagnostic value, GREM1, malignant, pulmonary nodules

## Abstract

To evaluate the diagnostic value of serum gremlin 1 (GREM1) levels in differentiating benign from malignant subsolid pulmonary nodules, a clinically challenging CT phenotype, this prospective observational cohort study included 183 patients with subsolid (ground‐glass) pulmonary nodules identified by thin‐section chest CT and 100 age‐ and sex‐matched healthy volunteers between January and December 2025. Patients were followed through the standard diagnostic pathway, and the final diagnosis was confirmed histopathologically. Serum GREM1 levels were quantified using ELISA. Receiver operating characteristic (ROC) curve analysis and multivariable binary logistic regression were used to evaluate diagnostic performance and factors associated with malignancy. Serum GREM1 levels increased stepwise from the healthy control group to the benign and malignant groups (all *p* < 0.05). The AUC of GREM1 for differentiating malignant nodules from benign nodules was 0.859, and the combined GREM1 and CYFRA21‐1 model achieved an AUC of 0.918. Multivariable analysis revealed maximum nodule diameter, serum GREM1 level, and CYFRA21‐1 level as independent risk factors associated with malignant nodules. Serum GREM1, particularly in combination with CYFRA21‐1, may improve differentiation between benign and malignant subsolid pulmonary nodules. Further studies with larger samples and external validation are needed.

## 1 Introduction

Lung cancer remains a major global public health burden, with an estimated 2.5 million new cases and 1.8 million deaths in 2022 [1, 2, 3]. Early detection is clinically meaningful because shifting diagnosis toward earlier stages enables curative‐intent management and translates into survival gains. Contemporary evidence from lung cancer screening studies supports this concept, with low‐dose CT screening demonstrating a reduction in lung cancer mortality and real‐world data linking screening to a favorable stage shift [4, 5]. With the widespread use of thin‐section CT, subsolid pulmonary nodules are being increasingly encountered. However, differentiating benign lesions from malignant lesions remains challenging in routine practice and often requires nuanced, individualized decision‐making [6, 7]. Therefore, identifying objective and minimally invasive biomarkers to support early benign–malignant discrimination of ground‐glass/subsolid nodules is still needed.

Blood‐based biomarkers have been increasingly explored as adjunct tools for distinguishing benign from malignant pulmonary nodules because they are minimally invasive and scalable and can provide complementary biological information beyond thin‐section CT, thereby supporting risk stratification and potentially reducing unnecessary invasive procedures in patients with indeterminate nodules [8, 9]. Gremlin 1 (GREM1) has been implicated in tumor‐related signaling networks and exhibits context‐dependent functions across cancer types [10, 11]. Mechanistically, GREM1 has been linked to key oncogenic processes, including epithelial‐mesenchymal transition, cancer‐associated fibroblast activation, angiogenesis, stromal remodeling, immune regulation, and therapeutic resistance, which together support its plausibility as a clinically informative circulating marker [12, 13]. In lung cancer, recent experimental work has further suggested that GREM1 participates in tumor‐promoting regulatory axes related to proliferation, invasion, and apoptosis [14]. However, clinical evidence focusing on serum GREM1 for differentiating benign from malignant subsolid pulmonary nodules remains limited, and its incremental value over conventional tumor markers in this specific diagnostic setting has not been well characterized.

In this context, we conducted a prospective observational cohort study to investigate the diagnostic value of serum GREM1 for differentiating benign from malignant subsolid pulmonary nodules. By providing clinical evidence for a minimally invasive biomarker in a high‐frequency yet diagnostically challenging CT phenotype, this study aims to support earlier risk stratification of ground‐glass nodules and inform individualized decision‐making for subsequent diagnostic work‐up and treatment planning.

## 2 Methods

### 2.1 Study Design and Participants

In this prospective observational cohort study, 183 adult patients with subsolid (ground‐glass) pulmonary nodules identified on thin‐section chest CT at our hospital between January 2025 and December 2025 were consecutively enrolled. Eligible nodules were pure ground‐glass nodules or part‐solid nodules, with a maximum diameter of 5–30 mm. Patients were followed through the standard diagnostic pathway, and the final classification of nodules as benign or malignant was determined on the basis of histopathological confirmation from surgical resection or CT‐guided biopsy specimens. Histopathological diagnoses were obtained by surgical resection in 147 patients and CT‐guided biopsy in 36 patients.

The inclusion criteria were as follows: (1) age ≥ 18 years; (2) at least one subsolid (ground‐glass) pulmonary nodule (pure GGN or part‐solid nodule) with a maximum diameter of 5–30 mm identified on thin‐section chest CT; and (3) availability of a definitive histopathological diagnosis (benign or malignant) obtained from surgical resection or biopsy specimens. The exclusion criteria were (1) a prior history of malignancy or current clinical/radiologic suspicion of metastatic pulmonary lesions; (2) any previous antitumor treatment for lung lesions, specifically including radiotherapy or chemotherapy; (3) evidence of acute infection or active systemic inflammatory disease at enrollment; (4) severe hepatic or renal dysfunction or other critical illness precluding study participation; and (5) incomplete clinical or imaging data. In addition, 100 age‐ and sex‐matched healthy volunteers were also recruited, and a single serum sample was collected as a reference control.

The study protocol was approved by the Ethics Committee of our hospital (Approval No. (2023) KLS (148)), and all procedures were conducted in accordance with the Declaration of Helsinki. Written informed consent was obtained from all participants prior to enrollment. All the data were de‐identified before analysis and used solely for research purposes.

### 2.2 Serum GREM1 Measurement

All participants underwent peripheral venous blood sampling in the morning after an overnight fast. Briefly, 5 mL of fasting antecubital venous blood was collected into serum tubes, allowed to clot at room temperature, and then centrifuged at 4°C to separate the serum. The serum supernatant was aliquoted into labeled cryovials and stored at −80°C until analysis, avoiding repeated freeze–thaw cycles. Serum GREM1, IL‐6, and IL‐1β concentrations were quantified using commercially available ELISA kits (MyBioSource, San Diego, CA, USA) according to the manufacturers' instructions, including GREM1 (MBS2020142; detection range 6.25–400 ng/mL), IL‐6 (MBS2021124; detection range 7.8–500 pg/mL), and IL‐1β (MBS2021180; detection range 15.6–1000 pg/mL). All samples were assayed in duplicate by investigators who were blinded to the clinical outcomes, and the mean values were used for subsequent analyses. In addition, the conventional serum tumor markers CEA, CYFRA21‐1, SCC, and NSE were measured as part of routine clinical testing in the hospital laboratory using standardized platforms. All measured GREM1, IL‐6, and IL‐1β concentrations were within the stated detection ranges. Therefore, no substitution, censoring, or dilution‐based remeasurement was required.

### 2.3 Clinical Data Collection

Clinical data were prospectively collected using a standardized case report form. Baseline information included age, sex, and body mass index (BMI), as well as smoking history (yes/no) and occupational/environmental exposure history (dust, asbestos, and cooking oil fumes). Comorbidities were documented at enrollment. Thin‐section chest CT data, including nodule multiplicity (solitary or multiple), lobar location (left upper lobe, left lower lobe, right upper lobe, right middle lobe, or right lower lobe), maximum nodule diameter (mm), nodule type, and consolidation‐to‐tumor ratio (CTR), were reviewed. First‐degree family history of cancer was recorded as yes or no. For malignant nodules, the Ki‐67 proliferation index (%), pathological T and N categories, and pathological stage were retrieved from pathology reports when available. Pathological staging was recorded according to the ninth edition of the TNM classification. The final diagnosis was established by histopathology, and the histological type was recorded for each case. Benign histological diagnoses included hamartoma, inflammatory lesions, granulomatous lesions, and fibrotic or scar‐related nodules. Malignant histological diagnoses included lung adenocarcinoma and lung squamous cell carcinoma.

### 2.4 CT Image Assessment

Thin‐section CT images were reconstructed at a slice thickness of 1.0 mm. Two thoracic radiologists, who were blinded to the histopathological diagnosis and serum GREM1 results, independently reviewed the images. The maximum diameters of the entire nodule and its solid component were measured on the image showing the extent of the largest lesion. The CTR was calculated as the maximum diameter of the solid component divided by the maximum diameter of the entire nodule, with pure ground‐glass nodules assigned a CTR of 0. Disagreements were resolved by consensus.

### 2.5 Statistical Analysis

Statistical analyses were performed using SPSS 25.0 (IBM, Armonk, NY, USA) and R version 4.4.0. Continuous variables were assessed for normality using the Shapiro–Wilk test and graphical inspection, and homogeneity of variance was assessed using Levene's test. Normally distributed data are presented as the means ± standard deviations and were compared between groups using Student's *t*‐test. Nonnormally distributed data are presented as medians (interquartile ranges) and were compared using the Mann–Whitney *U* test. Comparisons among three or more groups were performed using the Kruskal–Wallis test followed by Dunn's pairwise test with the Holm adjustment. Categorical variables are presented as number (percentage) and were compared using the chi‐square test. Spearman's rank correlation analysis was used to evaluate associations between serum GREM1 levels and continuous or ordinal variables. Receiver operating characteristic (ROC) curve analysis was performed to assess diagnostic performance, and areas under the ROC curves (AUCs) with 95% confidence intervals were reported. DeLong's test was used to compare the AUC of the GREM1 model with that of the combined GREM1 and CYFRA21‐1 model. The optimal cutoff value was determined by maximizing the Youden index. The resulting cutoff of 66.99 ng/mL was used to calculate the sensitivity, specificity, positive predictive value, negative predictive value, and positive and negative likelihood ratios, and 95% confidence intervals were calculated for sensitivity and specificity. A multivariable binary logistic regression model was constructed to identify independent factors associated with malignant nodules, and all the candidate variables were entered simultaneously using the enter method. No data were missing for the variables included in the analyses; therefore, complete‐case analysis was performed without imputation. Secondary subgroup, biomarker, and correlation analyses were considered exploratory. A two‐sided *p* < 0.05 was considered statistically significant.

## 3 Results

### 3.1 Comparison of Demographic and Clinical Characteristics Between Malignant and Benign Subsolid Pulmonary Nodule Groups

A total of 183 patients with subsolid pulmonary nodules, including 71 (38.8%) patients with malignant nodules and 112 (61.2%) patients with benign nodules, were enrolled. As shown in Table 1, age, sex distribution, BMI, smoking history, occupational/environmental exposure history, and comorbidities were comparable between the groups (*p* > 0.05). In terms of CT and diagnostic characteristics, compared with benign nodules, malignant nodules had a larger maximum diameter and a higher CTR (*p* < 0.05). In addition, some solid nodules accounted for a higher proportion and were more common in the malignant group than in the benign group (*p* < 0.05). Surgical resection was more frequently used for pathological confirmation in the malignant group, whereas CT‐guided biopsy was more common in the benign group (*p* < 0.05). Histopathological diagnosis was based on surgical resection in 147 patients (82 benign and 65 malignant) and CT‐guided biopsy in 36 patients (30 benign and 6 malignant). No patient had a documented interstitial lung disease.

**TABLE 1:** Clinical characteristics of patients with benign and malignant subsolid pulmonary nodules.

Variables	Benign, *n* = 112	Malignant, *n* = 71	*p*
Age (years)	53 (44–62)	53 (46–65)	0.258
BMI	22.38 ± 2.05	22.04 ± 2.31	0.299
Sex, male (%)	62 (55.4)	38 (53.5)	0.808
Smoking history (yes), *n* (%)	47 (42.0)	29 (40.8)	0.881
Occupational/environmental exposure history (yes)^a^, *n* (%)	32 (28.6)	12 (16.9)	0.072
Comorbidities, *n* (%)			
COPD	14 (12.5)	6 (8.5)	0.392
Diabetes mellitus	15 (13.4)	11 (15.5)	0.692
Hypertension	35 (31.3)	26 (36.6)	0.453
Coronary heart disease	7 (6.3)	9 (12.7)	0.134
Nodule multiplicity (multiple), *n* (%)	20 (17.9)	18 (25.4)	0.223
Lobar location, *n* (%)			0.997
Left upper lobe	29 (25.9)	19 (26.8)	
Left lower lobe	26 (23.2)	15 (21.1)	
Right upper lobe	19 (17.0)	13 (18.3)	
Right middle lobe	15 (13.4)	9 (12.7)	
Right lower lobe	23 (20.5)	15 (21.1)	
Maximum nodule diameter (mm)	10.15 ± 2.33	14.41 ± 3.31	< 0.001
Nodule type, *n* (%)			< 0.001
Pure ground‐glass nodule	77 (68.8)	17 (23.9)	
Part‐solid nodule	35 (31.2)	54 (76.1)	
Final histology (pathology), *n* (%)			
Hamartoma	31 (27.7)		
Inflammatory lesions	41 (36.6)		
Granulomatous lesions	16 (14.3)		
Fibrotic/scar‐related nodules	24 (21.4)		
Lung adenocarcinoma		61 (85.9)	
Lung squamous cell carcinoma		10 (14.1)	
Ki‐67, %		8 (5–12)	
Family history of cancer, *n* (%)	25 (22.3)	19 (26.8)	0.494
Pathological confirmation method, *n* (%)			0.002
Surgical resection	82 (73.2)	65 (91.5)	
CT‐guided biopsy	30 (26.8)	6 (8.5)	
Consolidation‐to‐tumor ratio	0.00 (0.00–0.08)	0.28 (0.06–0.52)	< 0.001

^a^ Occupational/environmental exposure history included exposure to dust, asbestos, and cooking oil fumes.

### 3.2 Serum Biomarker Levels in Malignant and Benign Nodules and Healthy Controls

Serum levels of GREM1, IL‐6, and IL‐1β, together with conventional tumor markers (CEA, CYFRA21‐1, SCC, and NSE), were compared among patients with malignant nodules, benign nodules, and age‐ and sex‐matched healthy volunteers (Figure 1). Serum GREM1 levels differed significantly across the three groups, showing a gradual increase from healthy controls to patients with benign nodules and malignant nodules. Holm‐adjusted Dunn tests revealed significant differences between healthy controls and benign nodules, between healthy controls and malignant nodules, and between benign and malignant nodules (all *p* < 0.05). Serum CYFRA21‐1 levels were significantly higher in the malignant group than in the benign and healthy control groups (both *p* < 0.05). Additionally, serum IL‐1β and IL‐6 levels were higher in both the malignant and benign groups than in the healthy control group (both *p* < 0.05). Because the distributional assumptions were not consistently satisfied, these comparisons were based on Kruskal–Wallis tests followed by Dunn's tests with the Holm adjustment.

**FIGURE 1:**
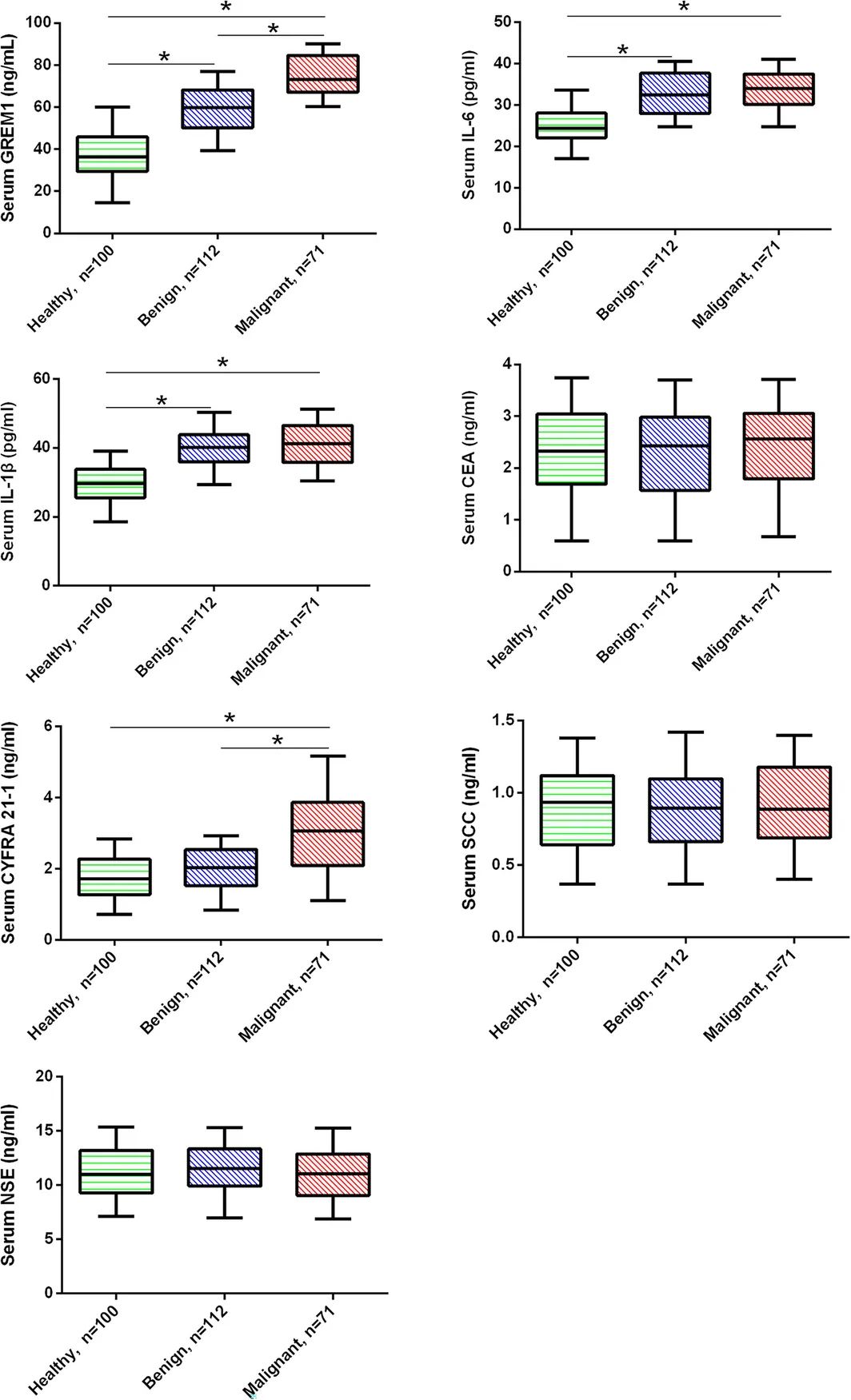
Serum levels of GREM1, IL‐6, IL‐1β, CEA, CYFRA21‐1, SCC, and NSE in healthy controls, patients with benign subsolid pulmonary nodules, and patients with malignant subsolid pulmonary nodules. Boxes represent the interquartile range, center lines indicate medians, and whiskers extend to 1.5 times the interquartile range. Group differences were assessed using the Kruskal–Wallis test followed by Dunn's test with the Holm adjustment. *p < 0.05.

### 3.3 Subgroup Analyses of Serum GREM1

Serum GREM1 differed among benign histological subgroups (Kruskal–Wallis *p* < 0.001), with median concentrations of 49.94 (43.62–56.50) ng/mL in hamartomas, 57.75 (51.45–64.75) ng/mL in inflammatory lesions, 67.60 (63.36–70.84) ng/mL in granulomatous lesions, and 68.14 (63.25–74.92) ng/mL in fibrotic/scar‐related nodules (Supplementary Table 1 and Supplementary Figure 1A). Holm‐adjusted Dunn tests revealed that serum GREM1 levels were significantly lower in hamartomas than in inflammatory lesions (*p* = 0.028), granulomatous lesions (*p* < 0.001), and fibrotic/scar‐related nodules (*p* < 0.001). Compared with granulomatous lesions (*p* = 0.028) and fibrotic/scar‐related nodules (*p* = 0.007), inflammatory lesions also had lower GREM1 levels, whereas no significant difference was observed between granulomatous and fibrotic/scar‐related nodules (*p* = 0.842). Among malignant nodules, serum GREM1 levels differed significantly among pathological T categories (Kruskal–Wallis *p* = 0.033; Supplementary Figure 1B). GREM1 expression was also positively correlated with pathological T category (*r* = 0.284; *p* = 0.016) and pathological stage (*r* = 0.317; *p* = 0.007); all malignant nodules were N0 and were classified as stage 0–IA3 (Supplementary Table 1). Serum GREM1 levels did not differ between lung adenocarcinoma and lung squamous cell carcinoma (*p* = 0.747; Supplementary Figure 1C). GREM1 was not associated with smoking status (*p* = 0.315) or a first‐degree family history of cancer (*p* = 0.244). Histology‐specific ROC analyses yielded an AUC of 0.862 (95% CI 0.809–0.915) for adenocarcinoma and 0.839 (95% CI 0.717–0.962) for squamous cell carcinoma. The squamous cell carcinoma result should be interpreted cautiously because this subgroup included only 10 patients (Supplementary Table 2 and Supplementary Figure 1D).

### 3.4 Correlation Analysis of Serum GREM1 With Clinical, CT, and Laboratory Variables

Spearman's rank correlation analysis was performed to evaluate the associations between serum GREM1 levels and predefined demographic, CT, and laboratory variables, including age, BMI, maximum nodule diameter, and the levels of IL‐6, IL‐1β, CEA, CYFRA21‐1, SCC, and NSE (Table 2). Serum GREM1 levels were positively correlated with maximum nodule diameter (*r* = 0.256; *p* < 0.001) and CYFRA21‐1 expression (*r* = 0.267; *p* < 0.001). Serum GREM1 was also positively correlated with the CTR in all patients with nodules (*r* = 0.392; *p* < 0.001) and in the part‐solid subgroup (*r* = 0.285; *p* = 0.007).

**TABLE 2:** Spearman's rank correlation analysis of serum GREM1 levels with demographic, CT, and laboratory variables.

Variables	GREM1	
	Spearman's correlation	*p*
Age	0.001	0.996
BMI	0.035	0.636
Maximum nodule diameter	0.256	< 0.001
CTR	0.392	< 0.001
IL‐6	0.119	0.109
IL‐1β	0.070	0.349
CEA	−0.004	0.956
CYFRA21‐1	0.267	< 0.001
SCC	0.020	0.793
NSE	−0.058	0.433

### 3.5 ROC Curve Analysis of Serum GREM1 Alone and in Combination With CYFRA21‐1

Serum GREM1 levels showed good diagnostic performance for differentiating malignant subsolid pulmonary nodules from benign ones, with an area under the curve (AUC) of 0.859 (95% CI: 0.808–0.910; *p* < 0.001; Figure 2). The optimal cutoff value was 66.99 ng/mL, which yielded a sensitivity of 76.1% and a specificity of 71.4%. A combined diagnostic model incorporating GREM1 and CYFRA21‐1 was subsequently established, and the predicted probability derived from this model was used for ROC analysis (Figure 2). The combined model further improved the diagnostic performance, with an AUC of 0.918 (95% CI: 0.876–0.959; *p* < 0.001). DeLong's test revealed that the AUC of the combined model was significantly greater than that of GREM1 alone (AUC difference = 0.059, 95% CI 0.018–0.101; *p* = 0.005). At the 66.99 ng/mL cutoff, the sensitivity was 76.1% (95% CI 65.0%–84.5%), the specificity was 71.4% (95% CI 62.5%–79.0%), the positive predictive value was 62.8%, the negative predictive value was 82.5%, the positive likelihood ratio was 2.66, and the negative likelihood ratio was 0.34.

**FIGURE 2:**
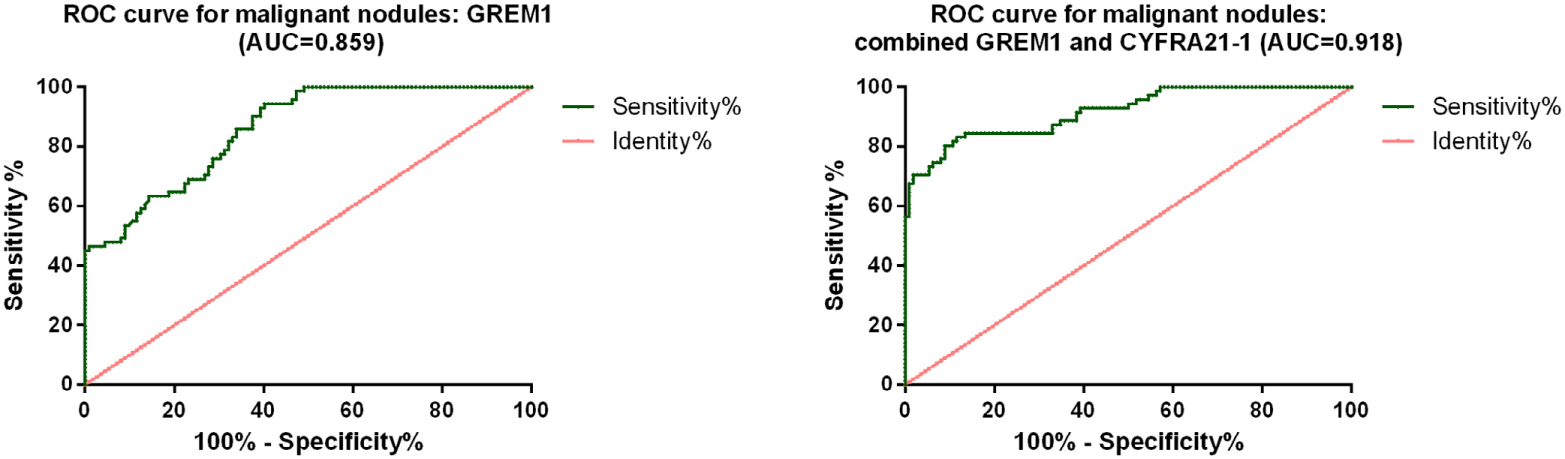
Receiver operating characteristic curve analysis for differentiating malignant from benign subsolid pulmonary nodules. The combined model had a significantly greater AUC than GREM1 alone according to DeLong's test (*p* = 0.005).

In an exploratory analysis defining obvious GREM1 elevation as ≥ 66.99 ng/mL, ROC analysis revealed a CTR cutoff of 0.10. The AUC was 0.677 (95% CI 0.604–0.750), with a sensitivity of 58.1% and a specificity of 72.2%. Because the discrimination was modest, this value should not be regarded as a definitive clinical CTR threshold.

### 3.6 Multivariable Binary Logistic Regression Analysis for Risk Factors for Malignant Nodules

A multivariable binary logistic regression model was constructed to identify independent factors associated with malignant subsolid pulmonary nodules, and all the candidate variables were entered simultaneously using the enter method (Table 3). The variables included age, BMI, sex, smoking history, occupational/environmental exposure history, COPD, diabetes mellitus, hypertension, coronary heart disease, nodule multiplicity, lobar location, maximum nodule diameter, nodule type, and serum biomarkers. In the multivariable analysis, maximum nodule diameter (OR = 2.773; 95% CI: 1.675–4.590; *p* < 0.001), serum GREM1 level (OR = 1.270; 95% CI: 1.106–1.457; *p* = 0.001), and CYFRA21‐1 level (OR = 8.881; 95% CI: 2.563–30.775; *p* = 0.001) were independently associated with malignant subsolid pulmonary nodules. In contrast, age, BMI, sex, smoking history, occupational/environmental exposure history, COPD, diabetes mellitus, hypertension, coronary heart disease, nodule multiplicity, lobar location, nodule type, and IL‐6, IL‐1β, CEA, SCC, and NSE levels were not significantly associated with malignancy in the adjusted model (all *p* > 0.05).

**TABLE 3:** Multivariable binary logistic regression analysis of factors associated with malignant subsolid pulmonary nodules.

Variables	Odds ratio	95% CI	*p*
Age	1.035	0.950–1.126	0.432
BMI	0.822	0.525–1.288	0.393
Sex	6.206	0.693–55.552	0.103
Smoking history	5.208	0.673–40.282	0.114
Occupational/environmental exposure history	0.246	0.024–2.490	0.235
COPD	0.145	0.001–30.823	0.481
Diabetes mellitus	4.939	0.316–77.269	0.255
Hypertension	0.732	0.112–4.767	0.744
Coronary heart disease	0.124	0.003–4.646	0.259
Nodule multiplicity (multiple)	4.917	0.541–44.697	0.157
Lobar location	0.781	0.386–1.578	0.491
Maximum nodule diameter	2.773	1.675–4.590	< 0.001
Nodule type	0.704	0.128–3.869	0.687
GREM1	1.270	1.106–1.457	0.001
IL‐6	1.150	0.957–1.381	0.135
IL‐1β	0.913	0.796–1.048	0.197
CEA	2.214	0.567–8.651	0.253
CYFRA21‐1	8.881	2.563–30.775	0.001
SCC	15.471	0.391–612.284	0.144
NSE	0.795	0.549–1.153	0.226

## 4 Discussion

Subsolid nodules detected on thin‐section CT are clinically common yet highly heterogeneous, and their slow growth and variable radiological pathology correspondence often complicate timely benign‐malignant discrimination and risk‐stratified management decisions [15, 16]. In this background, our prospective cohort showed a stepwise increase in serum GREM1 levels from healthy controls to patients with benign nodules to those with malignant nodules. GREM1 correlated with nodule diameter and CYFRA21‐1 and demonstrated diagnostic value for malignant pulmonary nodules, whereas the combined GREM1 + CYFRA21‐1 model further improved diagnostic performance. In addition, after multivariable adjustment, GREM1 remained an independent risk factor for malignant pulmonary nodules. Additional analyses revealed that GREM1 expression varied among benign histological subgroups, was positively associated with the CTR and early pathological T category, and was not associated with smoking status or family history of cancer.

Serum biomarkers are being increasingly investigated as adjunct tools for indeterminate pulmonary nodules because they provide systemic signals that can complement morphology‐based assessment and potentially improve early risk stratification in a minimally invasive manner. Serum CST1 was reported to be markedly elevated in NSCLC patients compared with benign pulmonary nodules and healthy controls, and a panel combining CST1 with CEA and CYFRA21‐1 achieved high discrimination for early‐stage NSCLC [17]. Circulating lncRNA THRIL also showed good discriminatory ability between benign and malignant solitary pulmonary nodules, and mechanistic assays supported a THRIL miR 99a‐IGF1R axis that promoted malignant phenotypes [18]. In screening‐detected nodules, radiomics alone achieved good classification performance, whereas serum metabolomics provided only a modest and statistically nonsignificant gain, highlighting that not every blood‐based layer improves an already strong imaging model [19]. With respect to SCLC, serum CDK1 was elevated compared with that in NSCLC pulmonary nodules and controls and shows strong differential diagnostic performance, and combining CDK1 with ProGRP and NSE further improved accuracy while correlating with stage and nodal metastasis [20]. Consistent with the broader literature showing that blood‐based markers can support risk stratification for indeterminate nodules but often require careful positioning alongside established tools, our results suggest that serum GREM1 provides an additional malignancy‐oriented signal beyond nonspecific inflammatory readouts, which may help refine the diagnostic workup of subsolid nodules.

Existing evidence suggests that circulating GREM1 has already been explored as a clinically relevant blood biomarker in several disease contexts, which supports its plausibility as a measurable and potentially actionable serum signal. In pancreatic ductal adenocarcinoma, serum GREM1 was reported to be elevated and to add diagnostic value when combined with CA19‐9, and higher serum GREM1 was also associated with poorer postoperative survival [21]. In interstitial lung disease, circulating GREM1 can capture clinically meaningful fibrotic biology [22]. Circulating GREM1 has also been reported to be elevated in systemic sclerosis, further supporting its potential utility as a noninvasive biomarker in human disease [23]. Building on these translational precedents, our findings support the practical application of serum GREM1 as an adjunct test in patients with indeterminate pulmonary nodules, where GREM1 may be used together with CYFRA21‐1 to improve discrimination and retain clinical relevance after adjustment for key covariates. The elevation of GREM1 expression in benign nodules compared with that in healthy controls may therefore partly reflect inflammatory and fibrotic remodeling because the values of granulomatous and fibrotic/scar‐related nodules were greater than those of hamartomas. These findings also indicate that GREM1 is not completely tumor‐specific. Because circulating GREM1 has been associated with interstitial lung disease, its diagnostic specificity may be reduced in patients with fibrotic lung disease. No patient in the present cohort had documented interstitial lung disease, and the performance of GREM1 in this population requires separate validation.

Mechanistically, GREM1 is a secreted BMP antagonist that can perturb BMP/SMAD signaling and thereby favor pro‐tumor phenotypes such as enhanced proliferation and invasive behavior in lung cancer models [24, 25]. By binding and sequestering BMP ligands, GREM1 may suppress canonical BMP receptor‐mediated SMAD1/5/9 activation and weaken BMP‐dependent differentiation signals. This imbalance may facilitate epithelial–mesenchymal transition, enhance tumor cell motility and invasion, and contribute to the progression of lung adenocarcinoma [25]. Beyond its role in BMP antagonism, GREM1 has been reported to bind and activate VEGFR2 in endothelial cells, thereby promoting VEGFR2‐dependent angiogenic responses in vitro and in vivo [26]. However, a subsequent study failed to reproduce GREM1‐mediated VEGFR2 phosphorylation in endothelial cells, suggesting that this noncanonical pro‐angiogenic activity may depend on the experimental system and cellular context [27]. In the tumor microenvironment, GREM1 was strongly expressed in cancer‐associated fibroblasts isolated from both primary NSCLC tissues and metastatic lymph nodes. These fibroblasts exhibited a myofibroblast‐like phenotype and enrichment of epithelial–mesenchymal transition‐related transcriptional programs, supporting a potential role for GREM1‐positive fibroblasts in fibroblast activation, extracellular matrix deposition, and stromal remodeling [28]. Moreover, knockdown of GREM1 in NSCLC‐derived cancer‐associated fibroblasts reduced M2 macrophage marker expression, suggesting that stromal GREM1 may contribute to the establishment of an immunosuppressive tumor immune microenvironment [28]. Another NSCLC study revealed that cancer‐associated fibroblast‐derived exosomal GREM1 activated an FGF4/SHH feedback loop in macrophages, promoted their polarization toward an immunosuppressive phenotype, and facilitated premetastatic niche formation [29]. Consistent with these mechanistic lines of evidence, the elevation of serum GREM1 in our malignant nodule group may reflect the combined contributions of tumor cells and activated stromal compartments, and after adjustment for key clinical covariates, GREM1 remained independently associated with malignant pulmonary nodules.

From a clinical perspective, our findings suggest that serum GREM1 could be considered an adjunct biomarker to support the evaluation of indeterminate pulmonary nodules, particularly subsolid lesions in which imaging‐based risk estimation may be uncertain. A blood‐based test is minimally invasive and can be repeated over time, which may be useful for longitudinal surveillance and for prioritizing patients who warrant closer follow‐up or earlier diagnostic procedures. Biologically, GREM1 and CYFRA21‐1 may reflect partly distinct but related components of malignant nodule biology. GREM1 is a secreted BMP antagonist associated with cancer‐associated fibroblast activation, extracellular matrix and stromal remodeling, angiogenesis, and regulation of the tumor immune microenvironment, whereas CYFRA21‐1 is a circulating fragment of epithelial cytokeratin 19 that can be released during tumor cell turnover and apoptosis [30]. Therefore, the weak positive correlation observed in our cohort (*r* = 0.267) may indicate that both biomarkers increase with malignant activity but provide only partially overlapping information. GREM1 may reflect predominantly stromal and tumor‐microenvironment remodeling, whereas CYFRA21‐1 more directly reflects epithelial tumor‐cell injury and turnover. Previous studies of pulmonary nodules have similarly shown that CYFRA21‐1 can improve malignancy discrimination when combined with circulating miRNAs, other serum proteins, clinical variables, or radiomic features [31, 32, 33]. Thus, combining GREM1 with CYFRA21‐1 may improve diagnostic performance by integrating stromal and epithelial signals that are not fully redundant, thereby providing a more comprehensive assessment of malignancy‐related biology in subsolid pulmonary nodules. Nevertheless, these results should be interpreted cautiously because the optimal cutoff may vary across populations and platforms, and clinical utility would require prospective external validation, calibration, and assessment of how biomarker‐informed decisions affect downstream management and outcomes.

This study has several limitations. First, this was a single‐center prospective study with a relatively small sample size, which may limit the generalizability of the findings. Second, serum GREM1 levels were measured at one point, which limits insight into its temporal behavior in nodule evolution. Third, although multivariable adjustment was performed, residual confounding related to unmeasured clinical or radiological factors cannot be fully excluded. Fourth, mechanistic inferences were based on prior literature rather than direct experimental validation within this cohort. Finally, external validation and standardized assay calibration are needed before clinical implementation can be considered, and the incremental value of GREM1 should be assessed within integrated diagnostic pathways rather than as a standalone test. In addition, all malignant nodules were N0 and Stage 0–IA3, which limits extrapolation to advanced lung cancer. The squamous cell carcinoma subgroup included only 10 patients, resulting in imprecise subgroup estimates. Finally, patients with documented interstitial lung disease were not represented, so the diagnostic performance of GREM1 in this population remains uncertain.

## 5 Conclusion

In conclusion, the results of this prospective study suggest that serum GREM1 levels are greater in patients with malignant pulmonary nodules than in those with benign nodules and in healthy controls and are associated with malignancy‐related nodule features. Serum GREM1 showed potential diagnostic value, and its combination with CYFRA21‐1 improved discrimination in our cohort. These findings indicate that GREM1 may serve as a useful adjunct biomarker to support the evaluation of indeterminate pulmonary nodules. Further multicenter studies with external validation and standardized testing are needed to clarify its clinical utility and to define how it may best be incorporated into existing diagnostic pathways.

## Funding

This study was supported by the Science and Technology Plan Project of Jiangxi Provincial Health Care Commission (202410096), Guiding Plan Project of Bureau of Science and Technology of Ganzhou (GZ2024ZSF260), Project of Bureau of Science and Technology of Ganzhou (GZ2024YLJ214), and Science and Technology Plan Project of Ganzhou Health Care Commission (GZWJW202402160).

## Ethics Statement

This study was approved by the Ethics Committee of Ganzhou Cancer Hospital (No. (2023) KLS (148)).

## Conflicts of Interest

The authors declare no conflicts of interest.

## Supporting information


**Supplementary Figure 1.** Subgroup analyses of serum GREM1 levels. (A) GREM1 levels among benign histological subgroups. (B) GREM1 levels according to pathological T category. (C) GREM1 levels in lung adenocarcinoma and lung squamous cell carcinoma. (D) Histology‐specific receiver operating characteristic curves for GREM1. Boxes represent the interquartile range, center lines indicate medians, and whiskers extend to 1.5 times the interquartile range.

**Supplementary Table 1.** Serum GREM1 levels according to histological and clinicopathological subgroups.
**Supplementary Table 2**. Histology‐specific ROC analyses of serum GREM1.

## Data Availability

All data generated or analyzed during this study are included in this article. The datasets used and/or analyzed during the current study are available from the corresponding author on reasonable request.
