# Supplementary material for: Diagnostic Value of Serum GREM1 Levels in Differentiating Benign and Malignant Subsolid Pulmonary Nodules: A Prospective Observational Study
Source: Kaohsiung J Med Sci. 2026 Nov 9:e70315. Online ahead of print. doi: 10.1002/kjm2.70315 (PMC13746230; doi:10.1002/kjm2.70315)
Supplement: Supplementary file 1 [file KJM2-9999-e70315-s001.docx]

# Supplementary Tables

Supplementary Table 1. Serum GREM1 levels according to histological and clinicopathological subgroups.

| **Analysis** | **Category** | **n** | **GREM1, median (IQR), ng/mL** | **p** |
| --- | --- | --- | --- | --- |
| Benign histology | Hamartoma | 31 | 49.94 (43.62–56.50) | <0.001 |
| Benign histology | Inflammatory lesion | 41 | 57.75 (51.45–64.75) |  |
| Benign histology | Granulomatous lesion | 16 | 67.60 (63.36–70.84) |  |
| Benign histology | Fibrotic/scar-related nodule | 24 | 68.14 (63.25–74.92) |  |
| Malignant histology | Lung adenocarcinoma | 61 | 73.17 (67.43–84.67) | 0.747 |
| Malignant histology | Lung squamous cell carcinoma | 10 | 76.51 (66.37–83.69) |  |
| Pathological T category | Tis | 8 | 68.04 (64.46–71.57) | r=0.284, p=0.016 |
| Pathological T category | T1mi | 12 | 76.60 (67.83–85.73) |  |
| Pathological T category | T1a | 17 | 70.84 (65.84–82.38) |  |
| Pathological T category | T1b | 26 | 73.03 (67.88–83.74) |  |
| Pathological T category | T1c | 8 | 84.88 (81.13–86.99) |  |
| Pathological stage | Stage 0 | 8 | 68.04 (64.46–71.57) | r=0.317, p=0.007 |
| Pathological stage | IA1 | 29 | 73.14 (66.86–84.56) |  |
| Pathological stage | IA2 | 26 | 73.03 (67.88–83.74) |  |
| Pathological stage | IA3 | 8 | 84.88 (81.13–86.99) |  |
| Smoking status | No | 107 | 66.18 (55.32–72.06) | 0.315 |
| Smoking status | Yes | 76 | 66.60 (60.24–76.00) |  |
| Family history of cancer | No | 139 | 66.18 (56.22–72.97) | 0.244 |
| Family history of cancer | Yes | 44 | 68.04 (61.52–77.26) |  |

For benign histological subgroups, the p value is from the Kruskal–Wallis test. Dunn’s pairwise tests were adjusted using the Holm method. Pathological T category and stage were analyzed using exploratory Spearman correlations. Holm-adjusted Dunn pairwise comparisons for benign histological subgroups were as follows: hamartoma vs inflammatory lesion, p=0.028; hamartoma vs granulomatous lesion, p<0.001; hamartoma vs fibrotic/scar-related nodule, p<0.001; inflammatory lesion vs granulomatous lesion, p=0.028; inflammatory lesion vs fibrotic/scar-related nodule, p=0.007; and granulomatous lesion vs fibrotic/scar-related nodule, p=0.842.

Supplementary Table 2. Histology-specific ROC analyses of serum GREM1.

| **Malignant subtype** | **Cases** | **Benign controls** | **AUC** | **95% CI** | **Sensitivity at 66.99 ng/mL** | **Specificity** |
| --- | --- | --- | --- | --- | --- | --- |
| Adenocarcinoma | 61 | 112 | 0.862 | 0.809–0.915 | 78.7% | 71.4% |
| Squamous cell carcinoma | 10 | 112 | 0.839 | 0.717–0.962 | 60.0% | 71.4% |
